# Why are iron chelators not as effective as artemisinin in killing malaria parasites?

**DOI:** 10.1186/s13071-026-07373-6

**Published:** 2026-05-13

**Authors:** Jun Sun, Chuantao Fang, Wenwen Si, Xixi Qin, Fei Wang, Yanna Li, Jia Sun

**Affiliations:** 1 Institute for Infectious Diseases and Vaccine Development, School of Medicine, Tongji University, Shanghai, People’s Republic of China; 2 Shanghai Tenth People’s Hospital, Tongji University, Shanghai, People’s Republic of China; 3 Henan University of Chinese Medicine, 156 Jinshui East Road, Zhengzhou, 450008 People’s Republic of China

**Keywords:** Artemisinin, Free radical, Iron chelator, Iron utilization, *Plasmodium*, Dihydroartemisinin, Desferrioxamine, Heme

## Abstract

**Background:**

Artemisinin resistance endangers current artemisinin combination therapy (ACT), necessitating new ACT development. Artemisinin kills malaria parasites by generating free radicals via heme/iron activation, while iron chelators disrupt iron utilization. The combination of these two mechanisms may form a better antimalarial therapy. It is known that iron chelators have significantly weaker antimalarial effects than artemisinin, but the mechanism is unclear. Thus, clarifying this reason is essential for elucidating artemisinin’s antimalarial action and developing strategies to enhance ACT.

**Methods:**

We assessed the effects of artemisinin derivatives (dihydroartemisinin, DHA; artemether, ATM) and the iron chelator desferrioxamine (DFO) on parasite infection rate and morphology in vitro/in vivo. Single-cell RNA sequencing was used to compare *Plasmodium falciparum* 3D7’s sensitivity to DHA/DFO at 3, 9, and 24 h post-treatment, analyzing differential gene expression and affected functions. Transmission electron microscopy (TEM) was used to observe artemisinin’s impact on parasites.

**Results:**

Although all developmental stages of *P. falciparum* 3D7 exhibited sensitivity to 24-h DHA treatment, parasite counts at 12–30 h postinfection (hpi) decreased more rapidly following 9-h DFO treatment than 9-h DHA treatment. Notably, DHA upregulated iron utilization-related genes at 3 h post-treatment (hpt), whereas DFO did not. DHA and DFO exerted distinct effects on gene expression, particularly in parasites at 12–30 hpi, where DHA induced the expression of genes related to ribosome biogenesis and protein translation pathways. In functional assays, DFO reduced *P. falciparum* parasitic infection in vitro but failed to inhibit *P. yoelii* proliferation in vivo; the combination of ATM and DFO was less potent than ATM monotherapy. TEM observations revealed that ATM localized to the parasites’ digestive vacuoles and disrupted heme aggregation.

**Conclusions:**

Artemisinin and its derivatives exhibit more potent antimalarial activity than iron chelators, likely stemming from their ability to accumulate in the parasite’s digestive vacuoles, interact more effectively with heme and iron, and thereby disrupt heme/iron homeostasis and utilization.

**Graphical abstract:**

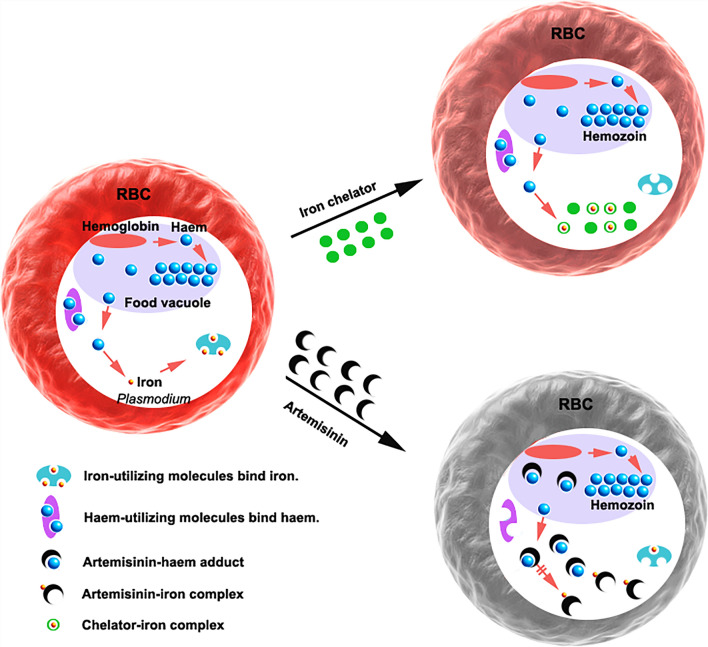

**Supplementary Information:**

The online version contains supplementary material available at 10.1186/s13071-026-07373-6.

## Background

Malaria remains a life-threatening parasitic disease, with the 2025 World Malaria Report documenting 282 million cases and an estimated 610,000 deaths [1]. Currently, artemisinin-based combination therapy (ACT) is the most effective treatment, yet prior antimalarial agents—including quinine, mepacrine, chloroquine, sulfadoxine-pyrimethamine, and mefloquine—have all succumbed to resistance [2, 3]. A pivotal shift in malaria control occurred in 2005 with the adoption of ACT, which pairs an artemisinin derivative with a longer-acting partner drug [4]. More recently, triple artemisinin-based combination therapy has emerged to address ACT resistance [5–7], but a critical unresolved question remains: how to select optimal partner drugs to maximize therapeutic efficacy.

Extensive research has established that iron and heme play crucial roles in activating artemisinin and its derivatives [8–13]. This activation involves reductive cleavage of the peroxide bond, generating carbon-centered reactive radicals that kill parasites by alkylating essential proteins and peroxidizing membrane lipids [14]. Notably, iron chelators also exhibit antimalarial activity [15–22]: iron is indispensable for *Plasmodium* spp. growth, development, and reproduction [23–26], and chelators disrupt iron utilization to impair parasite survival. However, iron chelators consistently show weaker antimalarial efficacy than artemisinin, and this discrepancy could clarify the mechanisms underlying artemisinin’s potency.

To address this gap, we compared the impacts of the iron chelator desferrioxamine (DFO) and the artemisinin derivative dihydroartemisinin (DHA) on *P. falciparum* 3D7—including effects on developmental stages, parasite survival, and gene expression—in both in vitro and in vivo models, with the goal of deepening understanding of the antimalarial mechanism of ACT and optimizing the development of combination therapies.

## Methods

### In vitro culture of *Plasmodium falciparum*

*P. falciparum* 3D7 was obtained from the Chinese Center for Disease Control and Prevention. *Plasmodium falciparum* strain 3D7 was maintained in human O^+^ erythrocytes (hematocrit: 2%) in RPMI 1640 medium (Gibco, Grand Island, NY, USA) supplemented with 0.5% (w/v) Albumax I (Gibco), 0.2 mmol/L hypoxanthine (Sigma-Aldrich, St. Louis, MO, USA), 25 mmol/L HEPES (Gibco), 0.2% (w/v) NaHCO_3_, and 20 μg/mL gentamicin (Sigma-Aldrich). Cultures were incubated at 37 °C under a controlled atmosphere of 5% CO_2_, 5% O_2_, and 90% N_2_. Unsynchronized parasites were used for all in vitro assays, and parasitemia was monitored periodically via Giemsa-stained blood smears.

### Experimental animals

*Plasmodium yoelii* 17XL was obtained from the Chinese Center for Disease Control and Prevention. *P. yoelii* 17XL was maintained by serial passage in mice. Specific pathogen-free (SPF) grade Balb/c mice (6–8 weeks old, female, *n* = 10 per group) were purchased from Shanghai SLAC Laboratory Animal Co., Ltd. (Shanghai, China) and housed in the Laboratory Animal Center of Tongji University under controlled conditions: temperature (22 ± 2 °C), relative humidity (50 ± 5%), and a 12 h light/12 h dark cycle, with free access to standard rodent chow and sterile water. Mice were infected via intraperitoneal injection of (1–10) × 10^5^ *P. yoelii* 17XL-parasitized erythrocytes. Parasitemia was monitored daily via Giemsa-stained blood smears (stained for 10 min at room temperature, then rinsed with phosphate-buffered saline (PBS, pH 7.2)) and quantified by counting at least 2000 erythrocytes per smear under a light microscope (Motic PA53 FS6, Xiamen, China; 1000 × magnification). When parasitemia reached 15–20%, mice were used for in vivo assays.

### Effect of desferrioxamine (DFO) and dihydroartemisinin (DHA) on *P. falciparum* 3D7 in vitro

Asynchronized *P. falciparum* 3D7 cultures (initial parasitemia: 1.5–2%) were prepared as previously described [27], and divided into three groups (*n* = 3 independent experiments): (1) DHA-treated group: 5000 µL of culture treated with DHA to a final concentration of 200 nmol/L; (2) DFO-treated groups: 5000 µL of culture treated with DFO to final concentrations of 500 µmol/L or 1000 µmol/L (two separate subgroups); and (3) untreated control group: 5000 µL of culture without any drug addition. At 3, 9, and 24 hpt, 500 µL aliquots of parasite-infected erythrocytes from each group were mixed with 2000 µL RPMI 1640 medium and 25 µL Hoechst 33342 (Biyuntian, Cat# C1028; diluted 1:100 in RPMI 1640) for nuclear staining. Samples were incubated at 37 °C for 10 min in the dark, then washed twice with RPMI 1640 medium, resuspended in fresh medium, and analyzed via flow cytometry using a BD FACSAria II cell sorter (BD Biosciences). Parasite-infected erythrocytes were gated on the basis of 405 nm fluorescence (Hoechst 33342), with uninfected erythrocytes used as a gating control. All flow cytometry data were processed using FlowJo software, and the proportion of infected red blood cells (iRBC) among 100,000 counted cells was compared across groups.

Statistical analyses of group differences were performed using an unpaired *t*-test with GraphPad Prism 8.0 software; a *P* < 0.05 was considered statistically significant. Prior to flow cytometry, blood samples were collected to prepare thin smears. Smears were stained with Giemsa following standard protocols and examined using a light microscope (Motic, PA53 FS6).

### Cell isolation and cell sorting

After 0, 3, 9, and 12 h of treatment with dihydroartemisinin (DHA) and desferrioxamine (DFO), 3–5 drops of blood were collected from *P. falciparum* 3D7 cultures and transferred to 50 mL centrifuge tubes containing 10 mL of RPMI 1640 medium. All treated samples and untreated control samples were immediately placed on ice for cell sorting. Cell sorting was performed using a BD FACS-Aria II flow cytometer (BD Biosciences) equipped with UV (355 nm), blue (488 nm), and red (633 nm) lasers. Parasitized erythrocytes (positive cells) were sorted and collected into RPMI 1640 medium supplemented with 5% (w/v) bovine serum albumin (BSA). Immediately following sorting, the collected parasitized erythrocytes were processed for single-cell RNA sequencing (scRNA-seq) analysis.

### 10 × genomics chromium library preparation and sequencing

Single-cell suspensions were loaded onto the 10 × Genomics Chromium Controller platform (10 × Genomics, Inc., Pleasanton, CA, USA) for single-cell capture, targeting approximately 5000–10,000 individual cells per sample. All procedures followed the manufacturer’s standard protocols for the 10 × Genomics Chromium Single-Cell 3’ Library and Gel Bead Kit v3.1 (Product Number: PN-1000268 for 16 reactions; 10 × Genomics, Inc., Pleasanton, CA, USA), which includes the Gel Bead Kit, Library Construction Kit, and Chip Kit required for droplet-based single-cell encapsulation and barcoded cDNA generation. Subsequent steps, including cDNA amplification and library construction, were performed strictly in accordance with the kit’s operational workflow as detailed in the *Chromium Single Cell 3’ Reagent Kits User Guide* (v3.1 Chemistry, Document No. CG000315). The resulting qualified libraries were sequenced by LC-Bio Technology Co., Ltd. (Hangzhou, China) on an Illumina NovaSeq 6000 sequencing system, employing a multiplexed paired-end sequencing strategy with a read length of 150 bp.

### Single-cell RNA sequencing (scRNA-seq) analysis

Single-cell suspensions were loaded onto the 10 × Genomics Chromium Controller platform (10 × Genomics, Inc., Pleasanton, CA, USA) for droplet-based encapsulation, enabling the generation of barcoded cDNA from individual cells. scRNA-seq libraries were subsequently prepared following the manufacturer’s protocols outlined in the *10* × *Genomics Chromium Single Cell 3′ Reagent Kit v3 User Guide* (10 × Genomics, Inc.). Libraries were sequenced on an Illumina NovaSeq^TM^ 6000 system (Illumina, Inc., San Diego, CA, USA). Raw sequencing data were demultiplexed and converted to FASTQ format using Illumina bcl2fastq software (v2.17.1; Illumina, Inc.). Downstream analyses, including sample demultiplexing, barcode processing, and single-cell 3′ gene expression counting, were performed using Cell Ranger software (v7.0.0) (10 × Genomics, Inc.), a dedicated pipeline for 10 × Genomics single-cell data. scRNA-seq reads were aligned to the *Plasmodium falciparum* reference genome (Ensembl Protists; Assembly accession: GCA_000002765.3).

### Clustering and visualization

Unsupervised clustering was performed using the Seurat package (v4.1.0) in R, which enabled exploration of quality control (QC) metrics and cell filtering. We visualized gene and molecule counts and their relationships. Genes expressed in fewer than one cell were filtered out, and cells with abnormally high numbers of detected genes—suggestive of potential multiplets—were excluded. The specific filtering criteria for cells were as follows: gene counts > 200 per cell, UMI counts without upper limits, and removal of doublets using DoubletFinder. Gene expression values were normalized using the LogNormalize method via the “Normalization” function in Seurat. Subsequently, the coefficient of variation for genes was calculated using Seurat. Dimensionality reduction was conducted with principal component analysis (PCA), focusing on the 2000 most variable genes. A *k*-nearest neighbor graph was constructed using Euclidean distances in the space of the first 20 significant principal components. To mitigate batch effects, Harmony was applied on the basis of the PCA results. Clusters were identified by optimizing the modularity function, and clustering results were visualized using Uniform Manifold Approximation and Projection (UMAP).

### Marker gene identification

To establish the relationship between cell clusters identified via UMAP analysis and infection stages, we selected genes that are specifically highly expressed at 6 h, 12 h, 18 h, 24 h, 30 h, 36 h, and 42 hpi with red blood cells (RBCs) from the transcriptome data of *Plasmodium falciparum* 3D7 (GEO Series accession number: GSE150484; https://ncbi.nlm.nih.gov/geo/query/acc.cgi?acc=GSE150484) [28] (Supporting Information Table S1).

### Desferrioxamine (DFO) and artemether (ATM) in vivo assay

In the in vivo experiment, all mice were infected with (1–10) × 10^5^ *P. yoelii* 17XL-parasitized erythrocytes via intraperitoneal injection. Once the infection rate of *P. yoelii* 17XL reached 15–20%, the mice were randomly divided into four groups. Group 1 received artemether at a dose of 100 mg/kg. Group 2 received subcutaneous injections of deferoxamine mesylate (MedChemExpress, MCE) at 500 mg/kg every 3 h after the initial injection. Group 3 was treated with artemether (100 mg/kg) combined with subcutaneous injections of deferoxamine mesylate. Group 4 received subcutaneous injections of an equivalent volume of saline. Statistical analyses were performed using unpaired *t*-tests with GraphPad Prism 8.0 software. A *P* < 0.05 was considered statistically significant.

### Effect of iron addition on artemisinin action

The *P. falciparum* parasites (3D7 strain) were cultured as previously described [27]. To assess the impact of iron addition on dihydroartemisinin (DHA) activity against asynchronous *P. falciparum* 3D7 at 3, 9, and 24 h post-treatment, erythrocytes infected with *P. falciparum* 3D7 (parasitemia: 1.5–2%) were divided into three groups, with treatments as follows: (1) DHA was added to 5000 µL of asynchronous *P. falciparum* 3D7 culture to achieve a final concentration of 150 nmol/L; (2) Iron sucrose (200 µmol/L final concentration) and DHA (150 nmol/L final concentration) were coadministered to 5000 µL of asynchronous *P. falciparum* 3D7 culture; (3) An asynchronous *P. falciparum* 3D7 culture without any treatment served as the untreated control. Subsequently, 500 µL of parasite-infected erythrocytes were mixed with 2000 µL of RPMI 1640 medium and 25 µL of Hoechst 33342 (Biyuntian, Cat# C1028; 1:100 dilution) for nuclear staining, and the mixture was incubated at 37 °C for 10 min in the dark. After washing twice with RPMI 1640 medium, samples were resuspended in the same medium and analyzed via flow cytometry using a BD FACSAria II cell sorter (BD Biosciences). Parasite-infected erythrocytes were gated on the basis of the fluorescence signal at 405 nm (from Hoechst 33342), with uninfected erythrocytes used as a gating control. All flow cytometry data were processed using FlowJo software, and the proportion of infected red blood cells (iRBCs) among 100,000 total cells was compared across groups.

### Transmission electron microscopy (TEM) analysis of artemisinin’s effect on hemozoin formation

*P. falciparum* 3D7-infected erythrocytes were treated with 150 nmol/L dihydroartemisinin (DHA) for 3–9 h, then incubated with saponin at a final concentration of 0.15% and centrifuged at 10,000 rpm for 5 min. The resulting black pellet was collected for transmission electron microscope (TEM) analysis to assess artemisinin’s effect on hemozoin formation. Samples were fixed in 2.5% glutaraldehyde overnight at 4 °C, washed with phosphate-buffered saline (PBS, pH 7.2), and postfixed in 1% osmium tetroxide. They were dehydrated in acetone and embedded in Epon 812. Thin Sects. (60 nm thickness) were cut using an ultramicrotome (EM UC6, Leica, Germany), mounted on Formvar-coated copper slot grids, and stained with uranyl acetate and lead citrate. Samples were observed using a JEOL EW-1230 TEM (Japan), with images acquired via a digital photodocumentation system (Gatan Bioscan Camera, Model 792) [29, 30].

### Statistical analysis

All statistical analyses were performed using GraphPad Prism 8.0 and R software (> 4.0.0). Group differences in parasitemia and other outcomes were analyzed via unpaired *t*-tests (two-tailed *P* < 0.05 for significance). Parasite proportion comparisons between DHA and DFO treatments (same infection stage/time point) were conducted using R’s prop.test function, with significance levels indicated as *P < 0.01, **P < 0.001, ***P < 0.0001.

Flow cytometry data were processed with FlowJo software. Raw scRNA-seq data were converted to FASTQ via Illumina bcl2fastq (v2.17.1), and downstream analyses (gene counting, alignment to *P. falciparum* genome GCA_000002765.3) were performed using Cell Ranger (v7.0.0). scRNA-seq bioinformatics analyses in R (Seurat v4.1.0) included QC filtering (> 200 genes/cell, doublet removal via DoubletFinder), LogNormalize normalization, PCA (2000 most variable genes), Harmony batch correction, and UMAP visualization of cell clusters. Infection stage annotation was based on marker genes from Gene Expression Omnibus (GEO) dataset GSE150484.

## Results

### Differential effects of DHA and DFO on *P. falciparum* 3D7

**Fig. 1:**
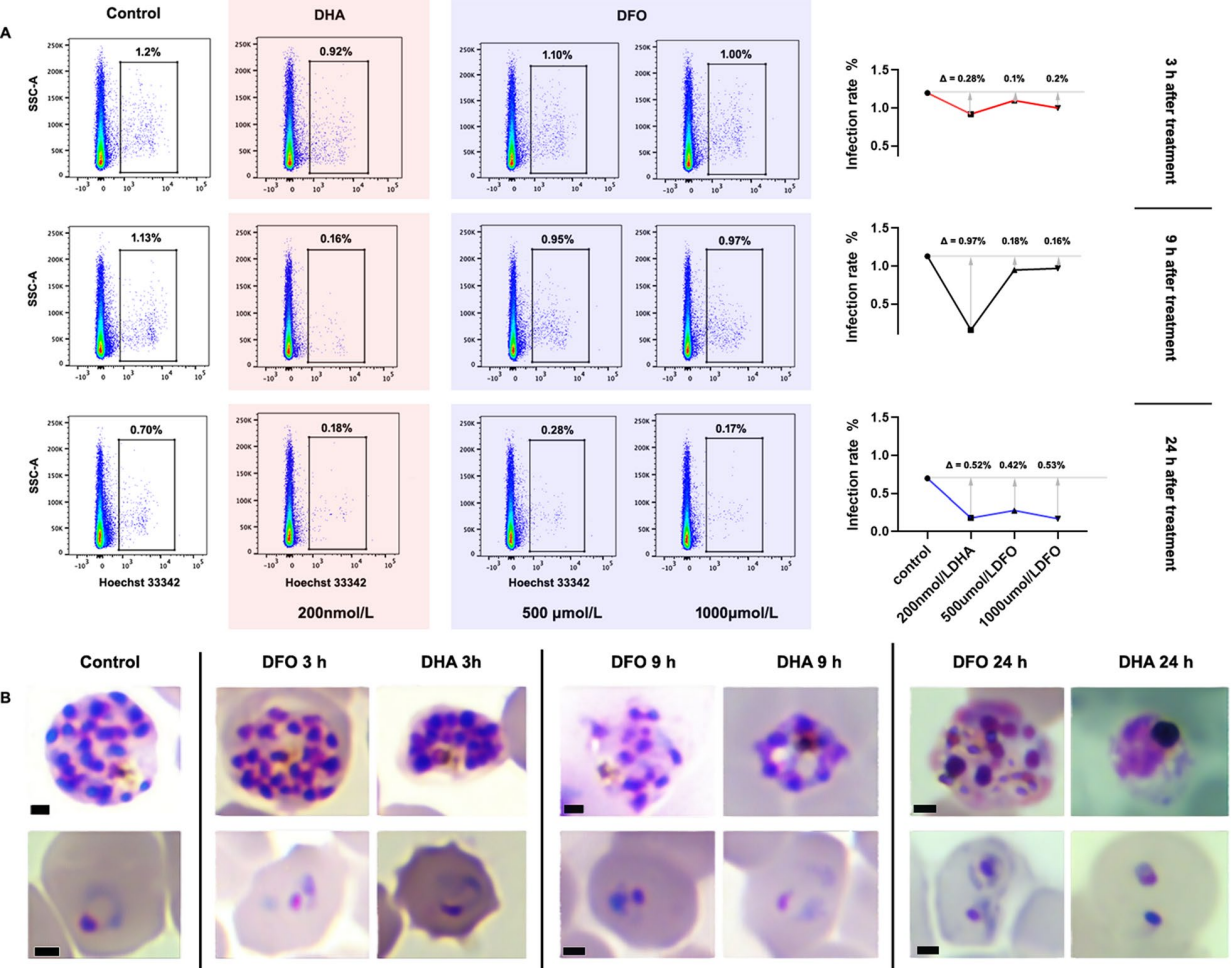
Effects of dihydroartemisinin (DHA) and desferrioxamine (DFO) on the morphology and infection rate of *P. falciparum* 3D7. **A** Flow cytometric analysis demonstrated different effects of DHA and DFO at various concentrations and exposure times. **B** Microscopic images showed the effects of DHA or DFO at varying concentrations and exposure times on *P. falciparum* 3D7. All scale bars represent 1 μm

Both dihydroartemisinin (DHA) and desferrioxamine (DFO) exert antimalarial activity against the *P. falciparum* 3D7 strain. As shown in Fig. 1A, time-course analysis of infection rates (with Δ defined as the reduction amplitude) revealed distinct inhibitory profiles between the two compounds. At 3 hpt, relative to the untreated control infection rate of 1.2%, the DHA group (200 nmol/L) exhibited a reduction to 0.92% (Δ = 0.28%). In contrast, DFO treatment at concentrations 2500-fold (500 µmol/L) and 5000-fold (1000 µmol/L) higher than DHA resulted in only modest reductions, with infection rates decreasing to 1.10% (Δ = 0.10%) and 1.0% (Δ = 0.20%), respectively. Both Δ values were lower than that of DHA. At 9 hpt, DHA-mediated inhibition was substantially enhanced, with the infection rate declining to 0.16% (Δ = 0.97%) relative to the untreated control of 1.13%. In sharp contrast, DFO showed minimal time-dependent improvement: the 500 µmol/L and 1000 µmol/L groups reduced infection rates to 0.95% (Δ = 0.18%) and 0.97% (Δ = 0.16%), respectively, with Δ values far lower than those observed for DHA. At 24 hpt, DHA maintained potent antimalarial activity, with the infection rate at 0.18% (Δ = 0.52%) relative to the untreated control of 0.70%. For DFO, a clear concentration-dependent enhancement of efficacy was observed: the 1000 µmol/L group achieved an infection rate of 0.17% (Δ = 0.53%), which was comparable to that of DHA (Δ = 0.52%). In contrast, the 500 µmol/L DFO group showed a moderate reduction to 0.28% (Δ = 0.42%) (Fig. 1A).

Morphologically, *P. falciparum* ring forms and schizonts exhibited differential responses to DHA and DFO treatment. At 3 and 9 hpt, both compounds induced varying degrees of structural damage to ring forms and schizonts. Notably, at 24 hpt, DHA caused significantly more severe structural damage to both developmental stages (ring forms and schizonts) than the DFO-treated group (Fig. 1B).

### Single-cell transcriptome analysis reveals effects of DFO and DHA on different infection stages of *P. falciparum* 3D7 at various time points

**Fig. 2:**
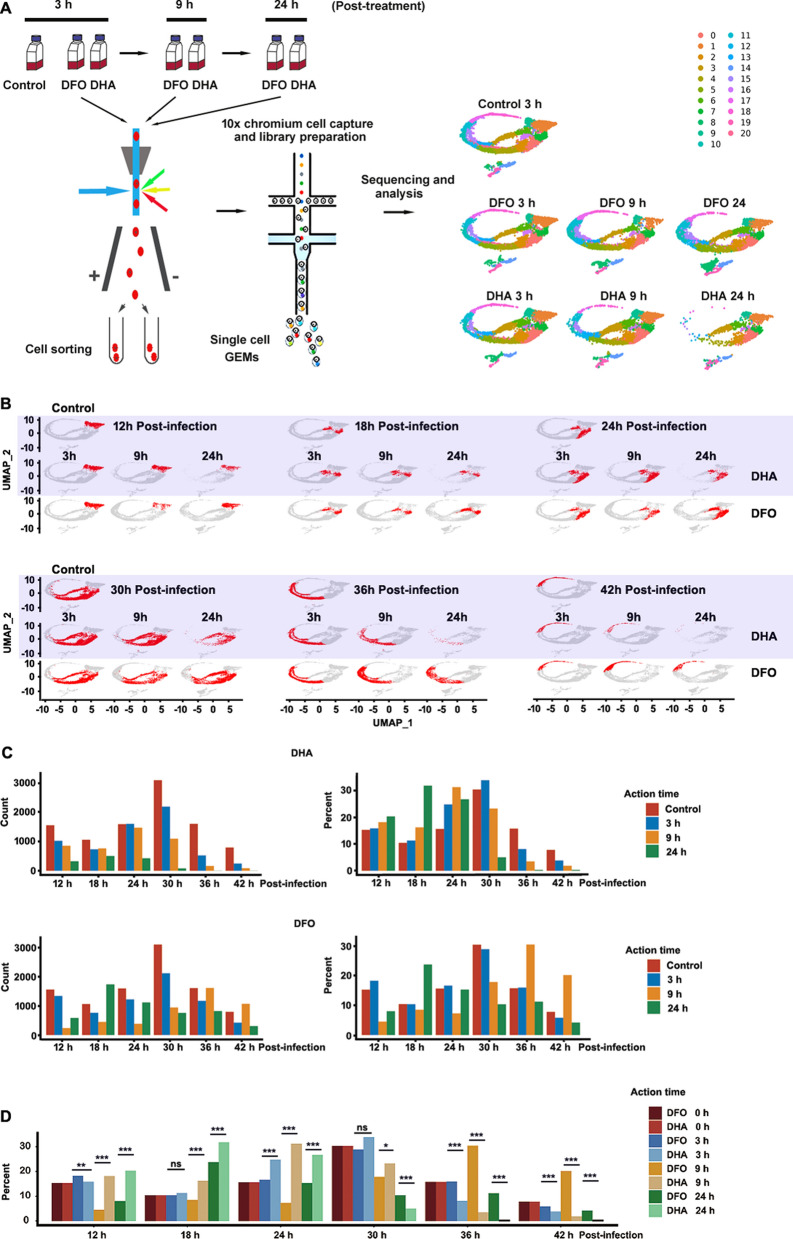
Uniform Manifold Approximation and Projection (UMAP) plots illustrating the effects of dihydroartemisinin (DHA) and desferrioxamine (DFO) on various infection stages of *P. falciparum* 3D7 at 3 h, 9 h, and 24 h post-treatment. **A** This panel shows the workflow of single-cell transcriptome sequencing, along with UMAP visualizations of single-cell transcriptomes from different samples. **B** UMAP plots reveal the effects of DHA and DFO on distinct infection stages of *P. falciparum* 3D7 at the indicated time points (3 h, 9 h, and 24 h post-treatment). Red color denotes parasites at the target infection stage, while grey color indicates parasites at other infection stages serving as the background. **C** Panel C presents alterations in the counts and proportions of parasites across distinct infection stages following 3 h, 9 h, or 24 h of exposure to DHA or DFO. Notably, the 30 hpi stage serves as a critical watershed: prior to this stage, parasites at 12 h, 18 h, and 24 hpi were more resistant to DHA than to DFO; beyond this juncture, parasites at 36 h and 42 hpi exhibited enhanced sensitivity to DHA. **D** A proportion test was performed using the prop.test function in R to analyze significant differences between DHA and DFO treatments at the same infection stage and the same post-treatment time point. Significance levels were indicated as *P < 0.01, **P < 0.001, and ***P < 0.0001

The distinct effects of DFO and DHA on different *P. falciparum* infection stages at 3 h, 9 h, and 24 h post-treatment were further analyzed using single-cell RNA sequencing (scRNA-seq) (Fig. 2A). After 3 h of treatment, neither compound caused evident damage across all parasite stages (Fig. 2B). However, proportional analysis indicated that DHA exerted stronger effects on parasites at 36 h and 42 hpi stages (Fig. 2C and D). In contrast, following 9 h of DFO treatment, more severe parasite damage was observed at 12 h, 18 h, and 24 hpi stages compared with the DHA-treated group. The 30 hpi stage represented a critical watershed: beyond this time point, parasites at the 36 h and 42 hpi stages displayed enhanced sensitivity to DHA. Notably, the proportion of parasites at the 36 h and 42 hpi stages following 9 h of DFO treatment exceeded 20%, while the corresponding proportion in the DHA-treated group remained below 5% (Fig. 2C and D). After 24 h of treatment, all parasite stages exhibited greater sensitivity to DHA compared with DFO (Fig. 2B). Consistent with the 9 h DFO treatment, proportional analysis confirmed that parasites at 12 h, 18 h, and 24 hpi sustained more severe damage in the DFO‑treated group relative to the DHA group, while parasites at 36 h and 42 hpi remained more sensitive to DHA (Fig. 2D).

### Gene expression changes associated with sensitivity to DHA and DFO

**Fig. 3:**
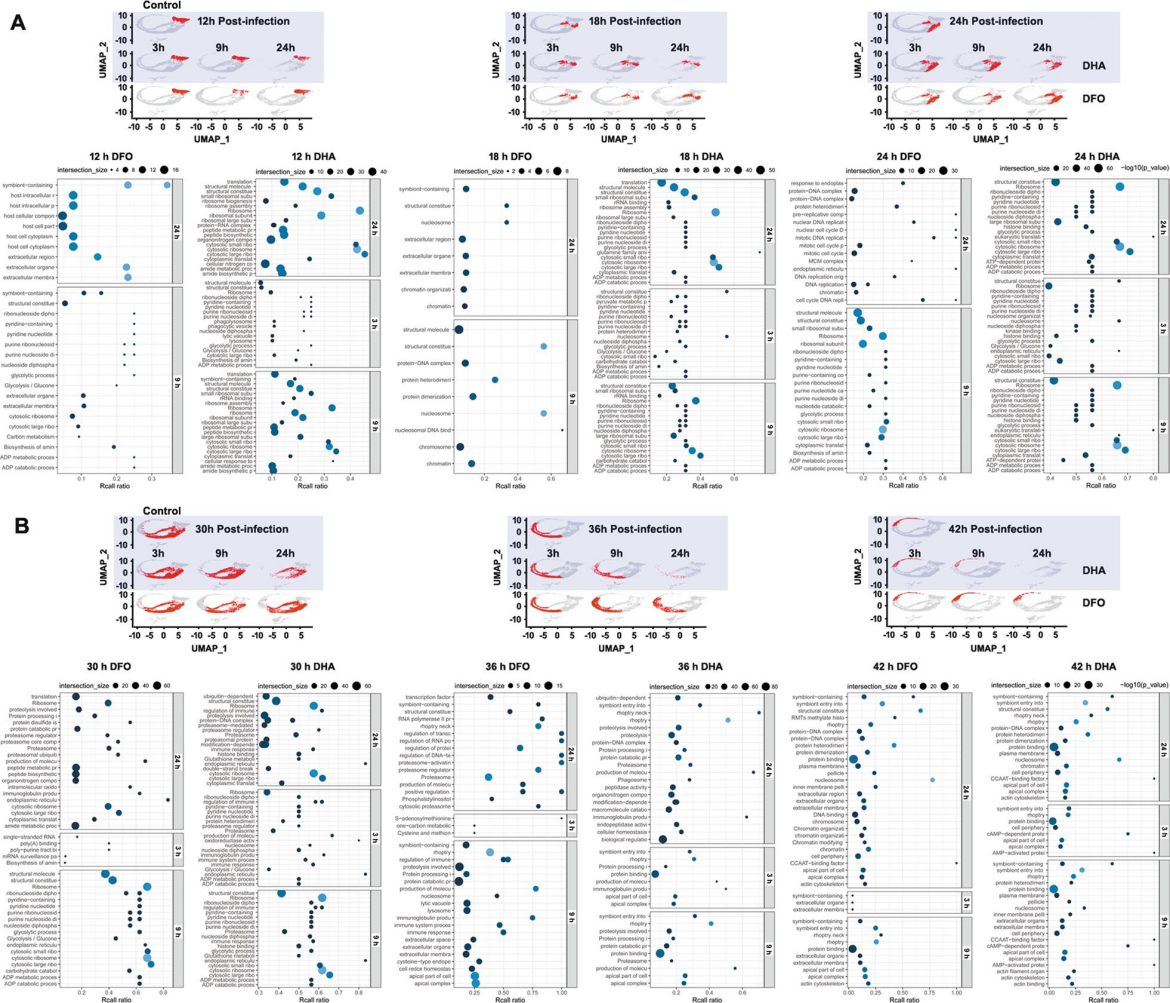
Functional analysis of differentially expressed genes across *P. falciparum* infection stages following 3 h, 9 h, or 24 h of treatment with DHA or DFO. **A** Gene Ontology (GO) enrichment analysis of differentially expressed genes (DEGs) at the 12 h, 18 h, and 24 h infection stages (see Additional file 1–3). **B** Gene Ontology (GO) enrichment analysis of DEGs at the 30 h, 36 h, and 42 h infection stages (see Additional file 4–6)

In parasites at 12 hpi, 3-h DFO treatment did not induce differential gene expression compared with the control group, whereas 3-h DHA treatment slightly altered functions related to ribosomes, pyridine metabolism, lysosomes, and purine metabolism. Notably, the functional changes induced by 3-h DHA treatment were similar to those caused by 9-h DFO treatment, including glycolysis, ADP metabolic processes, purine nucleoside diphosphate catabolic processes, pyridine nucleotide catabolic processes, symbiont-containing vacuole membrane functions, and gluconeogenesis. In contrast, 9-h DHA treatment significantly affected cytoplasmic translation, ribosome assembly, and peptide biosynthetic processes—effects that persisted with 24-h DHA treatment. By comparison, 24-h DFO treatment only impacted limited functions related to symbiont-containing vacuole membranes and extracellular organelles. These findings suggest that DHA-induced damage may enhance parasitic protein synthesis at 12 hpi as a repair mechanism to mitigate further injury, whereas parasites surviving DFO treatment appear to prioritize metabolic processes (Fig. 3A and Additional file 1).

In parasites at 18 hpi, 3-h DFO treatment still did not induce differential gene expression, while 3-h DHA treatment altered pyridine-containing compound catabolism, purine nucleoside diphosphate catabolism, and glycolysis—consistent with its effects on 12 hpi parasites. Additionally, 9-h and 24-h DHA treatments affected ribosome assembly, large ribosomal subunit biogenesis, and translation, mirroring their impacts at 12 hpi. In contrast, 9-h and 24-h DFO treatments significantly altered nucleosome structure, protein-DNA complex organization, chromatin dynamics, and chromosome functions—effects distinct from those of DHA (Supplementary Fig. 3A and Additional file 2).

In parasites at 24 hpi, 3-h DFO treatment remained without significant effect on gene expression, while 3-h DHA treatment induced changes comparable to those observed at 18 hpi. Similarly, 9-h and 24-h DHA treatments elicited responses consistent with their effects at 18 hpi. Notably, 9-h DFO treatment began to overlap with 9-h and 24-h DHA treatments, affecting functions such as ribosome assembly. A 24-h DFO treatment, however, uniquely enhanced differential expression of genes associated with the mitotic cell cycle, DNA replication, and protein-DNA interactions, distinct from DHA-induced changes (Supplementary Fig. 3A and Additional file 3).

In parasites at 30 hpi, 3-h DFO treatment affected only limited functions, including amino acid biosynthesis and the mRNA surveillance pathway. By contrast, 3-h DHA treatment influenced ribosome function, immune responses, purine/ribonucleoside diphosphate catabolism, endoplasmic reticulum lumen processes, nucleosome formation, glycolysis, and ribosome synthesis—consistent with earlier time points. The 9-h and 24-h DHA treatments similarly persisted in altering ribosome synthesis, protein-DNA complex assembly, proteasome activity, proteasomal protein catabolism, immune responses, cytoplasmic translation, peptide biosynthesis, and ribosome biogenesis. Notably, 9-h and 24-h DFO treatments now mirrored these DHA-induced effects, particularly in ribosome synthesis, translation, and peptide biosynthesis (Supplementary Fig. 3B and Additional file 4).

In parasites at 36 hpi, 3-h DFO treatment affected limited pathways: S-adenosylmethionine metabolism, cysteine/methionine metabolism, and one-carbon metabolism. In contrast, 3-h DHA treatment impacted protein binding, symbiont entry into the host, rhoptry function, apical complex organization, immune mediator production, and proteasome activity. The 9-h and 24-h DHA treatments elicited consistent responses, including effects on rhoptries, symbiont entry, host-interacting proteins, and proteasomes; 24-h DHA treatment additionally affected ubiquitin processes, protein-DNA interactions, post-translational modifications, and immune-related responses. The 9-h DFO treatment partially overlapped with these effects, influencing immune-related responses, symbiont-containing vacuole activities, and rhoptry/apical complex synthesis. However, 24-h DFO treatment uniquely altered transcription initiation regulation, protein catabolic processes, proteasomal activity regulation, and proteasome activation—distinct from DHA (Supplementary Fig. 3B and Additional file 5).

In parasites at 42 hpi, 3-h DFO treatment affected symbiont-containing vacuole formation and extracellular organelle function, while 3-h DHA treatment influenced symbiont entry into the host, protein binding, and rhoptry/apical complex functions, consistent with the effects of 9-h DFO and DHA treatments, which additionally involved the actin cytoskeleton and extracellular membranes. The 24-h DHA treatment primarily impacted these functions, including symbiont-containing vacuole formation, rhoptry/apical complex organization, protein-DNA interactions, protein binding, nucleosome dynamics, and actin cytoskeleton regulation. The 24-h DFO treatment, however, uniquely affected DNA binding, chromosome structure, and chromatin organization, distinct from DHA (Supplementary Fig. 3B and Additional file 6).

Collectively, parasites exhibited time-dependent and treatment-specific responses to DFO and DHA. At 12, 18, and 24 hpi, 3-h DFO treatment had minimal effects, whereas 3-h DHA treatment consistently induced differential gene expression. Particularly, by 9 h of treatment, parasites appeared more sensitive to DFO, with distinct expression patterns emerging between the two compounds: In DFO-treated groups: 12 hpi parasites showed altered expression of genes involved in host–parasite interactions, pyridine/purine metabolism, and glycolysis; 18 hpi parasites affected protein-DNA interactions, chromosome structure, and chromatin dynamics; 24 hpi parasites began to exhibit changes in ribosome and ribosomal subunit functions. In DHA-treated groups: 12, 18, and 24 hpi parasites consistently showed characteristic effects on ribosome synthesis and translation. Obviously, after 9 h of treatment with DFO and DHA, the former exhibits diverse multi-module functions (including chromatin regulation, symbiont-associated interactions, glycolysis, and basic protein synthesis) centered on glycolysis as the core metabolic pathway; in contrast, the latter is specifically focused on the entire protein synthesis process (ribosome assembly-translation), supplemented by cellular heat stress response, protein processing, and refined nucleotide catabolism. (Fig. 3A and Supplementary Table S2). At 30 hpi, the DFO and DHA treatment groups converged in their functional effects, both impacting ribosome synthesis and translation (Fig. 3B). Consistent with this observation, the two groups are centered on three core functional changes (ribosome-mediated protein synthesis, glycolysis-driven energy supply, purine nucleotide catabolism), share the endoplasmic reticulum lumen as a protein processing site, and align in the “protein synthesis-energy metabolism” pathway. (Fig. 3B and Supplementary Table S2). By 36 and 42 hpi, their effects partially overlapped, with both drugs impacting reproduction-related functions—yet they still retained distinct features. Neither compound significantly affected ribosome synthesis or translation, but both modulated genes associated with rhoptry formation, symbiont entry, and apical complex organization. Notably, DFO uniquely altered DNA binding, chromosome structure, and chromatin organization at these later time points (Fig. 3B).

### Expression changes of iron utilization-related genes in *P. falciparum* 3D7 following treatment with DFO and DHA

**Fig. 4:**
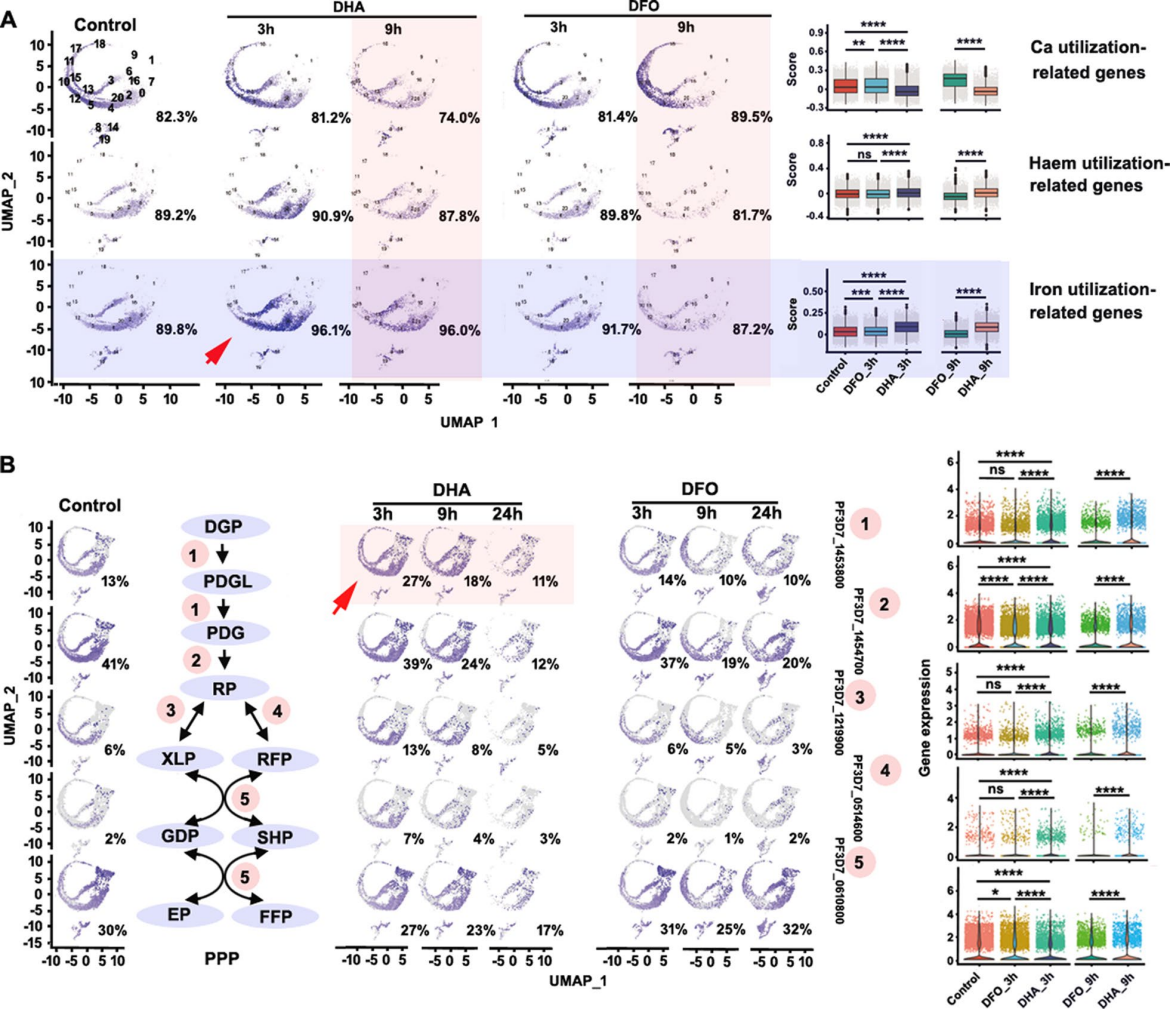
UMAP plots illustrating the effects of DHA and DFO on parasites expressing genes related to iron, heme, and calcium utilization, as well as genes associated with the pentose phosphate pathway (PPP). **A** UMAP plots show that after 3 h and 9 h of DHA treatment, the number of parasites expressing iron utilization-related genes was significantly higher than in the DFO-treated group. Each purple spot denotes a parasite expressing the target gene, with the purple intensity indicating the gene expression level (darker purple = higher expression; lighter purple = lower expression). Grey spots represent parasites not expressing the target gene. This proportion refers to the percentage of target gene-expressing parasites among all parasites. Boxplots reveal that 3 h- and 9 h-DHA treatment resulted in higher expression levels of iron utilization- and heme utilization-related genes compared with DFO treatment, in contrast to the expression pattern of calcium utilization-related genes (see Additional file 7). **B** UMAP plots reveal that after 3 h, 9 h, and 24 h of DHA treatment, two key changes were observed relative to the DFO-treated group: the number of parasites expressing the glucose-6-phosphate dehydrogenase-6-phosphogluconolactonase gene (PF3D7_1453800) increased significantly, and the expression level of this gene was also significantly upregulated. Additionally, ribulose-phosphate 3-epimerase (PF3D7_1219900) and ribose-5-phosphate isomerase (PF3D7_0514600) showed slight upregulation compared with the DFO-treated group. (*DGP* D-glucopyranose 6-phosphate, *PDGL* 6-O-phosphonato-D-glucono-1,5-lactone, *PDG* 6-phosphonatooxy-D-gluconate, *RP* D-ribulose 5-phosphate, *XLP* D-xylulose 5-phosphate, *RFP* D-ribofuranose 5-phosphate, *GDP* D-glyceraldehyde 3-phosphate, *SHP* sedoheptulose 7-phosphate, *EP* D-erythrose 4-phosphate, *FFP* beta-D-fructofuranose 6-phosphate) Boxplots further show that differences in the expression levels of glucose-6-phosphate dehydrogenase-6-phosphogluconolactonase (PF3D7_1453800), 6-phosphogluconate dehydrogenase (PF3D7_1454700), ribulose-phosphate 3-epimerase (PF3D7_1219900), ribose-5-phosphate isomerase (PF3D7_0514600), and transketolase (PF3D7_0610800) exist between DHA and DFO treatment at 3 h and 9 h post-treatment. Asterisks denote significance. *P < 0.05; **P < 0.01; ***P < 0.001; ****P < 0.0001

Artemisinin exerts parasiticidal effects after activation by iron or heme, whereas iron chelators (e.g., DFO) likely kill parasites by disrupting iron utilization. Both mechanisms are closely associated with iron; thus, we analyzed the expression of genes related to iron and heme utilization in *P. falciparum* 3D7 following 3 h or 9 h of DHA or DFO treatment (Fig. 4A). Notably, compared with the control group (baseline: 89.8% of parasites expressing iron utilization-related genes among all parasites), the proportion of parasites expressing these genes increased to 96.1% after 3 h of DHA treatment and 96.0% after 9 h of DHA treatment. In contrast, DFO treatment did not induce this increase: the proportions of parasites expressing the iron utilization-related genes among all parasites were 91.7% (3 h) and 87.2% (9 h), respectively. These data reveal that the expression levels of iron utilization-related genes in DHA-treated parasites were significantly higher than those in DFO-treated counterparts. Furthermore, a 3-h DHA exposure exerted negative effects on the expression of calcium utilization-related genes. Collectively, these observations indicate that DHA exerts a more specific and profound regulatory impact on iron utilization than DFO.

### The effect of DFO and DHA treatment on the pentose phosphate pathway (PPP)

In malaria parasites, hemoglobin is ingested and degraded, releasing heme; heme is further broken down by glutathione to liberate iron, which can activate the pentose phosphate pathway (PPP) [31]. To investigate the effects of DHA and DFO on the PPP, we analyzed both the proportion of parasites expressing PPP-related genes and the expression levels of these genes. Uniform manifold approximation and projection (UMAP) plots showed that the expression of glucose-6-phosphate dehydrogenase (G6PD) and 6-phosphogluconolactonase (GluPho)—two enzymes that catalyze the first two steps of the PPP and are essential for the survival of blood-stage *P. falciparum*—was stimulated and upregulated. Specifically, at 3 h and 9 h post-DHA treatment, the proportion of parasites expressing these two genes increased to 27% of the total parasite population, compared with 13% in the control group (Fig. 4B). This proportion was significantly higher than that observed in DFO-treated parasites.

Among other PPP-related genes, the expression levels of ribulose-phosphate 3-epimerase and ribose-5-phosphate isomerase were also slightly upregulated at 3 h and 9 h after DHA treatment (Fig. 4B). Together, these findings indicate that DHA exerts a more pronounced impact on the PPP of *P. falciparum* than DFO.

### In vivo antimalarial efficacy: iron chelators (DFO) versus artemether (ATM)

**Fig. 5:**
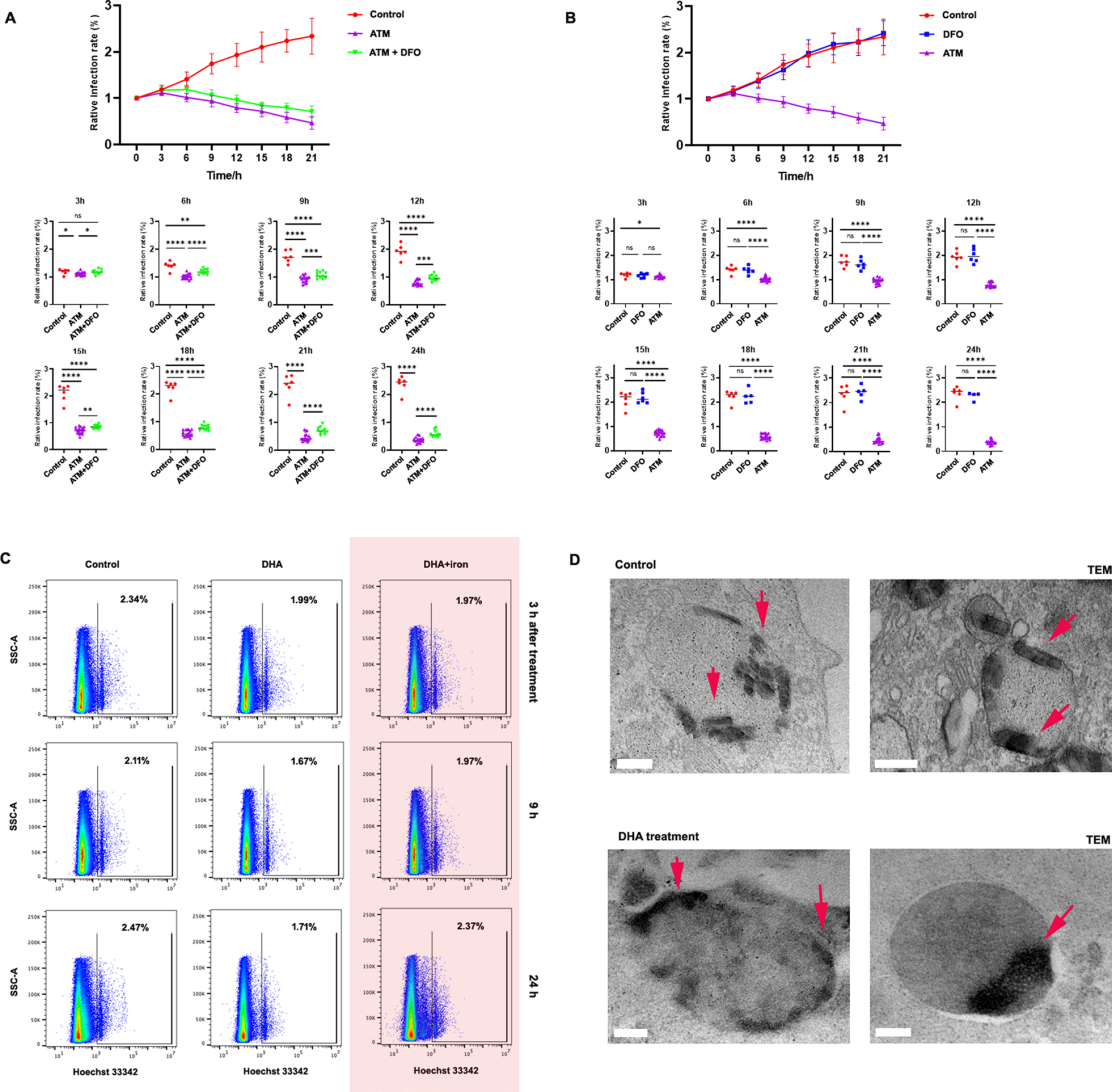
Effects of artemisinin and desferrioxamine (DFO) on malarial parasites in vivo and in vitro. **A** Effect of artemether alone and in combination with DFO on the infection rate of *Plasmodium yoelii* 17XNL (in vivo), revealing that DFO attenuates the antimalarial effect of artemether. **B** Effect of artemether or DFO on the infection rate of *P. yoelii* 17XNL (in vivo), demonstrating that artemether eliminates parasites, while DFO cannot do so. **C** Effect of iron supplementation on the infection rate of *P. falciparum* 3D7 in the presence of DHA (in vitro), revealing that iron addition attenuates the antimalarial effect of DHA—particularly following 9 h and 24 h of DHA treatment. **D** Transmission electron microscopy (TEM) images illustrate the effect of DHA on heme aggregation in lipid droplets within the digestive vacuole of *P. falciparum* 3D7, demonstrating DHA-induced damage to the structure of heme aggregates or hemozoin (red arrows). All scale bars represent 0.2 μm

To evaluate the in vivo antimalarial activity of iron chelators and artemisinin derivatives, we administered desferrioxamine (DFO) and artemether (ATM) to mice infected with *Plasmodium yoelii* 17XNL. Results showed that ATM induced a significant reduction in parasitemia, with its inhibitory effect strengthening over 21 h of observation (Fig. 5A). Interestingly, the combined administration of ATM and DFO also reduced parasitemia, but this effect was less potent than that of ATM alone. Despite both agents targeting free iron utilization, their combination failed to synergistically enhance the antimalarial efficacy of either single agent; instead, it may have exerted partial inhibitory or interfering effects. Notably, a key distinction emerged between the two agents: while DFO can kill malaria parasites in vitro, it was unable to eliminate parasites in vivo. In contrast, ATM achieved complete parasite clearance in the murine model (Fig. 5B). This limitation of DFO highlights a critical question for further investigation: why does DFO fail to eradicate malaria parasites in vivo, whereas ATM succeeds?

### In vitro investigation: impact of iron supplementation on dihydroartemisinin’s antimalarial efficacy

To explore the mechanism underlying the potent antimalarial effect of dihydroartemisinin (DHA) when interacting with iron, we analyzed how iron supplementation influences DHA’s efficacy against *Plasmodium falciparum* 3D7 at 3, 9, and 24 hpt (Fig. 5C). At 3 hpt, DHA (150 nmol/L) reduced parasitemia from 2.34% (control group) to 1.99%. The addition of iron sucrose (final concentration: 200 µmol/L) exerted a similar inhibitory effect to DHA alone, with no significant difference in parasitemia reduction. However, by 9 hpt, iron sucrose began to counteract DHA’s efficacy: DHA alone reduced parasitemia from 2.11% (control) to 1.67%, whereas cotreatment with iron sucrose increased parasitemia to 1.97%, weakening DHA’s inhibitory activity. This counteractive trend became more pronounced at 24 hpt: DHA alone lowered parasitemia from 2.47% (control) to 1.71%, while iron sucrose cotreatment elevated parasitemia to 2.37%—nearly restoring it to the control level. These findings indicate that iron supplementation reduces rather than enhances DHA’s antimalarial effect, challenging the current paradigm and strongly suggesting that additional mechanisms may synergistically contribute to DHA’s potent antimalarial activity.

### ARTs–heme–iron (HI) interaction in parasite digestive vacuoles

Iron chelators are water-soluble, whereas heme and artemisinin are lipid-soluble. Previous studies have demonstrated that heme accumulates in lipid droplets within the digestive vacuoles of malaria parasites [32–38]. We used transmission electron microscopy (TEM) to examine lipid droplets in *Plasmodium* spp. following treatment with DHA. At 24 h post-DHA treatment, TEM observations revealed distinct differences between the control and DHA-treated groups: in the control group, heme aggregated into regular crystal-like hemozoin structures along the membrane of lipid droplets. In contrast, DHA treatment disrupted this regular arrangement (Fig. 5D). These findings suggest that DHA can enter lipid droplets, interact with heme or hemozoin, and interfere with heme crystallization (i.e., hemozoin formation).

## Discussion

The emergence of artemisinin resistance has compelled the development of new antimalarial therapies, which in turn requires the identification of novel therapeutic targets. In our previous study, we highlighted the importance of hemozoin for malaria parasites: hemozoin likely plays a role in iron storage and the regulation of iron homeostasis [39], rendering hemozoin formation and iron utilization essential for parasitic survival.

It is well-established that artemisinin and its derivatives (ARTs) are activated by iron or heme to generate free radicals; these radicals alkylate parasitic proteins, induce peroxidation of critical membrane lipids, and trigger various other cellular perturbations [13, 40–44], ultimately leading to parasite death. In contrast, iron chelators (e.g., DFO) exert antimalarial effects by disrupting iron utilization. Given these complementary mechanisms, the combination of ARTs and iron chelators was initially hypothesized to be a promising strategy for developing new artemisinin-based combination therapies (ACTs). However, our findings challenge this notion, instead revealing potential limitations that must be addressed to advance more effective therapies.

Although iron chelators such as DFO can kill malaria parasites, their efficacy is limited and inferior to that of ARTs. To elucidate the underlying reasons, we compared gene expression profiles across different *Plasmodium* spp. developmental stages following treatment with DHA or DFO. Notably, the two treatments induced distinct patterns of differential gene expression: in parasites at 30 hpi and earlier stages, DHA treatment stimulated the expression of genes related to ribosome synthesis and protein translation; by 36 and 42 hpi, this transcriptional signature shifted to genes associated with merozoite-related structures and functions—including rhoptries, the apical complex, the apical region of the cell, and the actin cytoskeleton. In DFO-treated parasites, while some overlap with DHA-induced genes (e.g., merozoite-related functions) was observed at later stages, unique transcriptional changes were also evident, such as upregulation of genes involved in chromatin organization, protein-DNA complex assembly, nucleosome dynamics, regulation of RNA polymerase II-mediated transcription initiation, and regulation of protein catabolic processes.

Combined with morphological observations, these transcriptional data allow us to propose a model for parasitic responses to treatment: Following DHA exposure, parasites at early stages upregulate protein synthesis to repair DHA-induced damage—a compensatory mechanism that ultimately fails, as DHA causes irreversible, lethal injury. In contrast, DFO-treated parasites exhibit signs of attempted cellular recovery at later stages, such as enhanced transcription initiation and regulated protein catabolism. This distinction underscores why DHA-induced damage is fatal, whereas DFO’s effects are less potent and reversible.

Further insights into the mechanism of DHA’s superiority emerged from our analysis of iron-related gene expression: Even though DHA treatment promoted the expression of iron utilization-related genes, it exerted a stronger impact on iron utilization than DFO. Critically, iron supplementation assays revealed that increasing iron availability did not enhance DHA’s antimalarial efficacy—instead, it attenuated it. This finding suggests that DHA kills parasites not primarily by leveraging iron to generate free radicals, but rather likely by combining with additional mechanisms, such as interrupting iron utilization. Supporting this, previous studies have shown that ARTs can form adducts with heme [45–49]; we propose that these heme-ART adducts sequester heme, reduce the pool of free heme, and consequently decrease iron utilization, ultimately disrupting iron homeostasis and leading to parasite death.

Additionally, our transmission electron microscopy (TEM) observations align with this model: as lipid-soluble compounds, ARTs (e.g., DHA) can enter lipid droplets within the parasitic digestive vacuole—a phenomenon consistent with prior studies [33]—and disrupt the regular agregation of heme into hemozoin crystals. In contrast, water-soluble iron chelators including DFO cannot accumulate in lipid droplets or persist within parasites long enough to interact with heme/iron as effectively as ARTs. This difference in localization and retention likely explains why iron chelators are less potent than ARTs against malaria parasites.

## Conclusions

Interrupting iron utilization is a validated mechanism for eliminating malaria parasites, but ARTs exhibit unique and superior efficacy owing to their ability to interact with heme, disrupt hemozoin formation, and perturb iron homeostasis—effects that extend beyond the simple iron sequestration achieved by chelators. Importantly, our data show that combining ARTs with iron chelators does not enhance antimalarial activity in vivo; instead, it may even attenuate ART efficacy. Thus, when designing new ACTs targeting multiple parasitic pathways, it is advisable to avoid combining ARTs with agents that rely on iron utilization disruption. Instead, the focus should be on medications that damage *Plasmodium* through alternative mechanisms, thereby maximizing therapeutic synergy without risking interference with ART’s iron/heme-mediated mode of action.

## Supplementary Information

Additional file 1. Enrichment analysis of differentially expressed genes at 12 hours post-DHA and DFO treatment.

Additional file 2. Enrichment analysis of differentially expressed genes at 18 hours post-DHA and DFO treatment.

Additional file 3. Enrichment analysis of differentially expressed genes at 24 hours post-DHA and DFO treatment.

Additional file 4. Enrichment analysis of differentially expressed genes at 30 hours post-DHA and DFO treatment.

Additional file 5. Enrichment analysis of differentially expressed genes at 36 hours post-DHA and DFO treatment.

Additional file 6. Enrichment analysis of differentially expressed genes at 42 hours post-DHA and DFO treatment.

Additional file 7. Iron, heme and calcium utilization-related genes.

Additional file 8 . Marker genes highly expressed during different stages of *P. falciparum* 3D7 infection.

Additional file 9. Effect of 9 h DHA and DFO treatments on the gene expression profiles of parasites at distinct infection stages.

## Data Availability

Data supporting the main conclusions of this study are included in the manuscript.
