# Supplementary material for: Why are iron chelators not as effective as artemisinin in killing malaria parasites?
Source: Parasit Vectors. 2026 May 13;19:275. doi: 10.1186/s13071-026-07373-6 (PMC13340282; doi:10.1186/s13071-026-07373-6)
Supplement: Supplementary file 7 — Additional file 7. Iron, heme and calcium utilization-related genes. [file 13071_2026_7373_MOESM7_ESM.pdf]

Table. S1 Iron utilization-related genes of Plasmodium falciparum 3D7

Gene ID	Product Description
PF3D7_1223700	vacuolar iron transporter
PF3D7_0207200	iron-sulfur assembly protein, putative
PF3D7_0322500	iron-sulfur assembly protein, putative
PF3D7_0522700	iron-sulfur cluster assembly protein SufA
PF3D7_1103400	iron-sulfur cluster assembly protein SufD
PF3D7_1413500	iron-sulfur cluster assembly protein SufC
PF3D7_1454500	iron-sulfur cluster assembly protein ISCU
PF3D7_API04700	iron-sulfur cluster assembly protein SufB
PF3D7_0302700	CDGSH iron-sulfur domain-containing protein, putative
PF3D7_0416700	CDGSH iron-sulfur domain-containing protein, putative
PF3D7_0703100	cytosolic iron-sulfur assembly component 2, putative
PF3D7_1212800	succinate dehydrogenase [ubiquinone] iron-sulfur subunit, mitochondrial
PF3D7_1209400	cytosolic iron-sulfur protein assembly protein 1, putative
PF3D7_1439400	cytochrome b-c1 complex subunit Rieske, putative
PF3D7_1344600	lipoyl synthase
PF3D7_0623500	superoxide dismutase [Fe]
PF3D7_0814900	superoxide dismutase [Fe]
PF3D7_1022900	cytochrome c oxidase subunit ApiCOX13, putative
PF3D7_1342100	aconitate hydratase
PF3D7_0614200	cytosolic Fe-S cluster assembly factor NAR1, putative
PF3D7_0609100	zinc transporter ZIP1
PF3D7_1022300	ZIP domain-containing protein, putative
PF3D7_1361600	Fe-S assembly protein IscX, putative
PF3D7_1311000	protein ISD11
PF3D7_1318100	ferredoxin
PF3D7_1214600	adrenodoxin-type ferredoxin, putative
PF3D7_0930900	NifU-like protein, putative
PF3D7_0927300	fumarate hydratase
PF3D7_0720400	apoptosis-inducing factor, putative
PF3D7_0727200	cysteine desulfurase IscS
PF3D7_0921400	NifU-like scaffold protein
PF3D7_0614800	endonuclease III-like protein 1, putative
PF3D7_1128500	Fe-S cluster assembly factor HCF101, putative
PF3D7_1368200	ABC transporter E family member 1, putative
PF3D7_0824600	Fe-S cluster assembly protein DRE2, putative
PF3D7_0910800	cytosolic Fe-S cluster assembly factor NBP35, putative
PF3D7_0515800	BolA-like protein, putative
PF3D7_0905200	mitochondrial carrier protein, putative
PF3D7_1406900	radical SAM protein, putative
PF3D7_1464600	serine/threonine protein phosphatase UIS2, putative
PF3D7_1022800	4-hydroxy-3-methylbut-2-en-1-yl diphosphate synthase (ferredoxin)
PF3D7_0524900	S-adenosyl-L-methionine-dependent tRNA 4-demethylwyosine synthase, putative
PF3D7_0502800	DnaJ protein, putative
PF3D7_0920100	chaperone, putative
PF3D7_1452200	aminomethyltransferase, putative
PF3D7_0622200	tRNA-2-methylthio-N(6)-dimethylallyladenosine synthase
PF3D7_0907900	peptide deformylase
PF3D7_1227800	elongator complex protein 3, putative
PF3D7_0709200	glutaredoxin-like protein
PF3D7_0606900	glutaredoxin-like protein
PF3D7_0817100	tRNA modification GTPase, putative
PF3D7_0829400	prolyl 4-hydroxylase subunit alpha, putative
PF3D7_1435300	glutamate synthase [NADH], putative

PF3D7_0304500	1-cys-glutaredoxin-like protein-1
PF3D7_0910900	DNA primase large subunit, putative
PF3D7_0709000	chloroquine resistance transporter
PF3D7_0918000	glideosome-associated protein 50
PF3D7_0104400	4-hydroxy-3-methylbut-2-enyl diphosphate reductase
PF3D7_0511200	stearoyl-CoA desaturase
PF3D7_1017000	DNA polymerase delta catalytic subunit
PF3D7_0630300	DNA polymerase epsilon catalytic subunit A, putative

Table. S2 Haem utilization-related genes of Plasmodium falciparum 3D7

Gene ID	Product Description
PF3D7_1011900	heme oxygenase
PF3D7_1446800	heme detoxification protein
PF3D7_1203600	cytochrome c1 heme lyase, putative
PF3D7_1224600	cytochrome c heme lyase, putative
PF3D7_1462700	cytochrome c1, heme protein, mitochondrial, putative
PF3D7_0918100	cytochrome b5-like heme/steroid binding protein, putative
PF3D7_1428700	heme/steroid binding domain containing protein, putative
PF3D7_1364900	ferrochelatase
PF3D7_1246100	delta-aminolevulinic acid synthetase
PF3D7_1209600	porphobilinogen deaminase
PF3D7_0607300	uroporphyrinogen III decarboxylase
PF3D7_1232300	cytochrome b5, putative
PF3D7_0519300	protoheme IX farnesyltransferase
PF3D7_1440300	porphobilinogen synthase
PF3D7_1411700	methyltransferase, putative
PF3D7_1435000	cytochrome c oxidase assembly protein COX15, putative
PF3D7_MIT01400	cytochrome c oxidase subunit 3
PF3D7_1028100	protoporphyrinogen oxidase
PF3D7_1440200	stromal-processing peptidase, putative
PF3D7_1035400	merozoite surface protein 3
PF3D7_0716500	conserved Plasmodium protein, unknown function
PF3D7_0831800	histidine-rich protein II
PF3D7_MIT02100	cytochrome c oxidase subunit 1
PF3D7_1311700	cytochrome c, putative
PF3D7_1404100	cytochrome c, putative

Table. S3 Calcium utilization-related genes of Plasmodium falciparum 3D7

Gene ID	Product Description
PF3D7_0106300	calcium-transporting ATPase
PF3D7_0605400	calcium-binding protein, putative
PF3D7_1137700	calcium-binding protein, putative
PF3D7_0217500	calcium-dependent protein kinase 1
PF3D7_0310100	calcium-dependent protein kinase 3
PF3D7_0610600	calcium-dependent protein kinase 2
PF3D7_0717500	calcium-dependent protein kinase 4
PF3D7_1122800	calcium-dependent protein kinase 6
PF3D7_1123100	calcium-dependent protein kinase 7
PF3D7_1329700	apicoplast calcium binding protein 1
PF3D7_1337800	calcium-dependent protein kinase 5
PF3D7_1423600	calcium-dependent protein kinase, putative
PF3D7_0715300	calcium/calmodulin-dependent protein kinase, putative
PF3D7_1104900	calcium/calmodulin-dependent protein kinase, putative
PF3D7_1108600	endoplasmic reticulum-resident calcium binding protein
PF3D7_0816400	EF-hand calcium-binding domain-containing protein, putative
PF3D7_1444200	EF-hand calcium-binding domain-containing protein, putative
PF3D7_1464800	EF-hand calcium-binding domain-containing protein, putative
PF3D7_1211900	non-SERCA-type Ca2+ -transporting P-ATPase
PF3D7_1238900	protein kinase 2
PF3D7_0603500	cation/H+ antiporter
PF3D7_1362300	transmembrane and coiled-coil domain-containing protein 1, putative
PF3D7_0802800	serine/threonine protein phosphatase 2B catalytic subunit A
PF3D7_1351300	conserved Plasmodium membrane protein, unknown function
PF3D7_0511000	translationally-controlled tumor protein homolog
PF3D7_1465500	potassium channel K2
PF3D7_1030800	calmodulin, putative
PF3D7_1418300	calmodulin, putative
PF3D7_1110100	C2 domain-containing membrane protein
PF3D7_0702900	centrin, putative
PF3D7_1450000	serine/threonine protein kinase, putative
PF3D7_0926300	protein kinase, putative
PF3D7_0714400	calmodulin, putative
PF3D7_1446600	centrin-2
PF3D7_1352600	protein kinase, putative
PF3D7_1027700	centrin-3
PF3D7_1027700	centrin-3
PF3D7_1105500	centrin-4
PF3D7_0706100	EF hand domain-containing protein, putative
PF3D7_1434200	calmodulin
PF3D7_1250200	CSC1-like protein, putative
PF3D7_0517200	WD repeat-containing protein, putative
PF3D7_1243900	double C2-like domain-containing protein
PF3D7_1441300	serine/threonine protein kinase, putative
PF3D7_0321400	pseudo protein kinase 1, putative
PF3D7_0107000	centrin-1
PF3D7_0928900	guanylate kinase
PF3D7_1025000	Eps15-like protein
PF3D7_0414200	calmodulin-like protein

PF3D7_0414200	calmodulin-like protein
PF3D7_0627200	myosin light chain, putative
PF3D7_0110600	phosphatidylinositol-4-phosphate 5-kinase
PF3D7_1451700	calcineurin subunit B
PF3D7_0817500	histidine triad nucleotide-binding protein 1
PF3D7_1354800	metacaspase-1
PF3D7_0208100	C2 domain-containing protein 5, putative
PF3D7_1018200	pseudophosphatase PPP8
PF3D7_1313500	TMEM238 domain-containing protein, putative
PF3D7_1463900	rhoptry neck protein 11, putative
PF3D7_0712300	erythrocyte membrane protein 1, PfEMP1
PF3D7_0727800	cation transporting ATPase, putative
PF3D7_0904900	copper-transporting ATPase
PF3D7_0318900	PAT complex subunit CCDC47, putative
PF3D7_1362400	calpain
PF3D7_0408700	perforin-like protein 1
PF3D7_1417000	inner membrane complex protein 1l
PF3D7_1136300	tudor staphylococcal nuclease
PF3D7_1246400	myosin A-tail interacting protein
PF3D7_0926000	protein kinase, putative
PF3D7_0213400	protein kinase 7
PF3D7_1304100	DNA ligase I
PF3D7_1022700	phospholipid scramblase
PF3D7_1423300	serine/threonine protein phosphatase 7
PF3D7_0605300	aurora-related kinase ARK1
