# Supplementary material for: Why are iron chelators not as effective as artemisinin in killing malaria parasites?
Source: Parasit Vectors. 2026 May 13;19:275. doi: 10.1186/s13071-026-07373-6 (PMC13340282; doi:10.1186/s13071-026-07373-6)
Supplement: Supplementary file 8 — Additional file 8 . Marker genes highly expressed during different stages of P. falciparum 3D7 infection. [file 13071_2026_7373_MOESM8_ESM.pdf]

Table S1. Marker genes highly expressed during different stages of *P. falciparum* 3D7 infection.

6 -12h	18h	24h	30 h	36h	42 h
PF3D7_0936600					F3D7_0109000
	PF3D7_0601200	F3D7_0831400	PF3D7_1366500	PF3D7_0508000	
PF3D7_1035800					PF3D7_1030200

These marker genes were selected from the transcriptome data of *P. falciparum* parasite 3D7 strain https://ncbi.nlm.nih.gov/geo/query/acc.cgi?acc=GSE150484 (GEO Series accession number GSE150484).
