# Supplementary material for: Why are iron chelators not as effective as artemisinin in killing malaria parasites?
Source: Parasit Vectors. 2026 May 13;19:275. doi: 10.1186/s13071-026-07373-6 (PMC13340282; doi:10.1186/s13071-026-07373-6)
Supplement: Supplementary file 9 — Additional file 9. Effect of 9 h DHA and DFO treatments on the gene expression profiles of parasites at distinct infection stages. [file 13071_2026_7373_MOESM9_ESM.pdf]

Table S2. Effects of 9-h DHA and DFO treatments on the gene expression profiles and functions of parasites at distinct infection stages

Infection stages / Drugs	12 h post-infection	18 h post-infection	24 h post-infection	30 h post-infection	36h post-infection	42 h post-infection
DFO	GO:CC / symbiont-containing vacuole membrane<br>GO:CC / extracellular membrane-bounded organelle<br>GO:CC / symbiont-containing vacuole<br>GO:MF / structural constituent of ribosome<br>GO:BP / glycolytic process<br>GO:BP / ADP metabolic process<br>GO:BP purine nucleoside diphosphate catabolic process	GO:CC / nucleosome<br>GO:CC / chromatin<br>GO:CC / protein-DNA complex<br>GO:CC / chromosome<br>GO:MF / structural constituent of chromatin<br>GO:MF / protein heterodimerization activity<br>GO:MF / protein dimerization activity<br>GO:MF / structural molecule activity<br>GO:MF / nucleosomal DNA binding	GO:CC / cytosolic small **ribosomal** subunit<br>GO:CC / cytosolic **ribosome**<br>GO:CC / cytosolic large **ribosomal** subunit<br>GO:MF / structural constituent of **ribosome**<br>GO:MF / structural molecule activity<br>GO:BP / pyridine nucleotide catabolic process<br>GO:BP / purine nucleoside diphosphate catabolic process<br>GO:BP / ADP metabolic process<br>GO:BP / glycolytic process	GO:CC / cytosolic large **ribosomal** subunit<br>GO:CC / cytosolic small **ribosomal** subunit<br>GO:CC / cytosolic **ribosome**<br>GO:CC / endoplasmic reticulum lumen<br>GO:MF / structural constituent of **ribosome**<br>GO:MF / structural molecule activity<br>GO:BP / glycolytic process<br>GO:BP / ribonucleoside diphosphate catabolic process<br>GO:BP / pyridine nucleotide catabolic process<br>GO:BP / purine ribonucleoside diphosphate catabolic process	GO:CC / **nucleosome**<br>GO:CC / **rhoptry**<br>GO:CC / **apical complex**<br>GO:CC / **apical part of cell**<br>GO:CC / extracellular space<br>GO:CC / extracellular organelle<br>GO:MF / **cysteine-type endopeptidase activity**<br>GO:BP / production of molecular mediator of immune response<br>GO:BP / immunoglobulin production<br>GO:BP / regulation of immune system process<br>GO:BP / immune response	GO:CC / **rhoptry**<br>GO:CC / **apical complex**<br>GO:CC / **apical part of cell**<br>GO:CC / **actin cytoskeleton**<br>GO:CC / **symbiont-containing vacuole**<br>GO:CC / extracellular membrane-bounded organelle<br>GO:CC / extracellular organelle<br>GO:CC / **rhoptry neck**<br>GO:MF / protein binding<br>GO:BP / **symbiont entry into host**

DHA	GO:CC / cytosolic large **ribosomal** subunit<br>GO:CC / cytosolic ribosome<br>GO:CC / cytosolic small **ribosomal** subunit<br>GO:CC / small **ribosomal** subunit<br>GO:CC / **ribosomal** subunit<br>GO:MF / structural constituent of **ribosome**<br>GO:MF / rRNA binding<br>GO:MF / structural molecule activity<br>GO:BP / cellular response to heat<br>GO:BP / cytoplasmic translation<br>GO:BP / **ribosome** assembly<br>GO:BP / **ribosomal** large subunit biogenesis	GO:CC / cytosolic large **ribosomal** subunit<br>GO:CC / cytosolic **ribosome**<br>GO:CC / small **ribosomal** subunit<br>GO:MF / structural constituent of **ribosome**<br>GO:BP / pyridine-containing compound catabolic process<br>GO:BP / ADP metabolic process<br>GO:BP / ribonucleoside diphosphate catabolic process	GO:CC / eukaryotic translation elongation factor 1 complex<br>GO:CC / cytosolic large **ribosomal** subunit<br>GO:CC / endoplasmic reticulum lumen<br>GO:CC / cytosolic **ribosome**<br>GO:MF / histone binding<br>GO:MF / ATP-dependent protein folding chaperone<br>GO:MF / structural constituent of **ribosome**<br>GO:BP / purine ribonucleoside diphosphate catabolic process<br>GO:BP / ribonucleoside diphosphate catabolic process<br>GO:BP / ADP catabolic process	GO:CC / endoplasmic reticulum lumen<br>GO:CC / cytosolic large **ribosomal** subunit<br>GO:CC / cytosolic **ribosome**<br>GO:CC / cytosolic small **ribosomal** subunit<br>GO:MF / histone binding<br>GO:MF / structural constituent of **ribosome**<br>GO:BP / regulation of immune system process<br>GO:BP / regulation of immune response<br>GO:BP / immune response<br>GO:BP / purine ribonucleoside diphosphate catabolic process<br>GO:BP / glycolytic process	GO:CC / **rhoptry**<br>GO:CC / **apical part of cell**<br>GO:CC / **apical complex**<br>GO:MF / protein binding<br>GO:BP / **symbiont entry into host**<br>GO:BP / **proteolysis involved in protein catabolic process**<br>GO:BP / **protein catabolic process**<br>GO:BP / production of molecular mediator of immune response	GO:CC / **rhoptry**<br>GO:CC / **apical part of cell**<br>GO:CC / **apical complex**<br>GO:CC / **actin cytoskeleton**<br>GO:CC / **cAMP-dependent protein kinase complex**<br>GO:CC / **symbiont-containing vacuole**<br>GO:CC / extracellular membrane-bounded organelle<br>GO:CC / extracellular organelle<br>GO:CC / **symbiont-containing vacuolar space**<br>GO:MF / **actin binding**<br>GO:MF / protein heterodimerization activity<br>GO:MF / **AMP-activated protein kinase activity**<br>GO:BP / **symbiont entry into host**<br>GO:BP / **actin filament organization**

This table illustrates the impacts of 9-hour DHA and DFO treatments on parasite gene expression profiles, along with consequent structural/functional impairments and adaptive alterations, based on enriched GO terms.

- At **12–24 hpi**, DHA induced protein synthesis system perturbations, with enriched translation regulation and protein folding pathways (adaptive responses to counteract protein homeostasis damage). DFO disrupted host-parasite interaction and energy metabolism homeostasis, upregulating symbiont-containing vacuole, chromatin remodeling, and glycolysis pathways (hallmarks of iron deprivation-induced impairment).
- At **30 hpi**, both treatments triggered conserved adaptive responses, with comparable enrichment of core translation and nucleotide metabolic pathways, supporting basic survival amid treatment-induced damage.
- At **36–42 hpi**, despite structural/functional impairments, both treatments failed to eliminate parasite invasion-related machineries, as key invasive structures and pathways remained enriched, preserving partial invasion capability.
